# Outcome of gastroplasty and gastric bypass in a single centre in the UK

**DOI:** 10.1186/1756-0500-2-181

**Published:** 2009-09-13

**Authors:** T Okoro, M Sintler, A Khan

**Affiliations:** 1 School of Medical Sciences, University of Bangor, Penrallt Road, Bangor, Gwynedd, UK; 2 Department of Surgery, Sandwell Hospital, Birmingham, UK; 3 Department of Surgery, The Manor Hospital, Walsall, UK

## Abstract

**Background:**

Morbid obesity is defined as BMI>40 kg/m^2^. It affects 124,000 men and 412,000 women in England and Wales (NICE, July 2002). According to NICE guidelines, Bariatric surgery is indicated if the treatments for obesity such as exercise, diet and drugs fail. Procedures include laparoscopic gastric banding (LGB), vertical banded gastroplasty (VBG), and Gastric Bypass (GB).

**Aims:**

The aim of this audit was to determine if NICE guidelines on the use of Bariatric surgery in the Manor Hospital, Walsall was being adhered to. **Secondary aims were **also to establish if Bariatric surgery is achieving its goal in the long-term and if weight reduction is being maintained in this group of patients.

**Methods:**

A retrospective cohort study was carried out on patients who underwent Bariatric surgery between 1990 and 2004. Retrieved records were scrutinised and the following parameters were collated: pre-operative morbidities, intra and post-operative complication rates and weight reduction on follow-up.

**Results:**

129 patients were operated on in the 14 year period. For VBG, 40 out of 105 patients had weight gain by the 5th follow-up visit. This compared with 5 out of 18 patients after the same timescale for the GB group and 1 out of 6 in the LGB group. The most common post-operative complication was stenosis (28% of VBG group).

**Conclusion:**

Bariatric surgery is relatively safe as an intervention for morbid obesity. Weight loss however is not maintained in the long term. VBG and LGB are short term interventions. Further research is required to look into the merits of gastric bypass surgery.

## Background

Obesity is defined as a BMI (weight (kg)/height (m)^2^) greater than 30. The different classes of obesity are shown in Table 1[1].

**Table 1:** Categories of Obesity and NICE Guidelines (5) on Surgery for Morbid Obesity*

**Category**	**BMI (kg/m**^2^**)**
Underweight	<18.5

Normal	18.5-24.9

Overweight	25.0-29.9

Obesity Class I	30.0-34.9

Obesity Class II	35.0-39.9

Obesity Class III	>40

As Per NICE Guidelines, The individual	
• Must be aged 18 or over	
• Has to have been receiving treatment in a specialist obesity clinic as a hospital	
• Has tried all other appropriate non-surgical treatments to lose weight but have not been able to maintain weight loss	
• Has no specific medical or psychological reasons why they shouldn't have this type of surgery	
• Is generally fit enough to have an anaesthetic and surgery	
• Understands that they will need to be followed up by a doctor and other healthcare professionals such as dieticians or psychologists over the long term	

*Morbid Obesity = BMI >40 kg/m^2^

= BMI 35-40 kg/m^2 ^and significant disease that may be improved if weight loss is achieved (e.g. diabetes, hypertension)

In the UK in the last 10 years, the number of men defined as obese has increased by 75% (22.9% of the male population) whilst the number of women has also increased by a figure of 50% (23.5% of the female population) [2]. Morbid obesity is defined as a situation where an individual's BMI is greater than 40. Morbid obesity has been identified as a major risk factor for cardiovascular disease, hypertension, type II diabetes, cancer, psychological problems and sleep apnoea syndrome [3].

Diet and behavioural modifications have not been proven to be appropriately effective in the maintenance of weight loss over time. While several anti-obesity drugs are available, these rarely result in the loss of more than 10% of body weight [4]. At present, the only available therapeutic intervention that provides effective long term weight loss for the severely obese is Bariatric surgery [4].

According to NICE (National Institute for Clinical Excellence) guidelines in the UK, an individual can be offered Bariatric surgery if they fulfil the criteria as laid out in Table 1[5].

There are two main types of surgery available to aid weight reduction and these are known as 'malabsorptive' and 'restrictive'. Malabsorptive surgery works by shortening the length of the digestive tract so that the amount of food absorbed by the body is reduced. This type of surgery involves creating a bypass by joining one part of the intestine to another [6].

Restrictive surgery limits the size of the stomach so that the individual feels full after eating a small amount of food. This type of surgery can involve stapling parts of the stomach together (vertical band gastroplasty), or fitting an adjustable tight band to make a small pouch for food to enter (laparoscopic gastric banding) [4]. Gastric bypass employs both restriction and malabsorption in the aim of weight loss.

Bariatric surgery is not carried out as extensively in the UK as it is in the US where it is more established. The aim of this study was to investigate the short, medium and long term outcome of Bariatric interventions carried out at the Walsall Manor Hospital in the West Midlands between 1990 and 2004.

## Methods

Retrospective single centre audit of all Bariatric procedures carried out between 1990 and 2004 at the Manor hospital, Walsall. This is a district general hospital serving a population of approximately 275,000. Patient demographics, the primary procedure undertaken, side effects as well as secondary procedures undergone were recorded.

No ethical approval was required for this study. Audit approval was sought and obtained from the Local hospital audit department. No experimental research was performed during the course of this study. The treatment methods assessed are well recognised and established methods of Bariatric surgery.

### Operative technique

During the vertical banding gastroplasty (VBG), the fundus of the stomach was stapled parallel to the lesser curve using a surgical stapling device. The distal exit of the created pouch was then narrowed with a band. A food receiving reservoir of ~50-60 ml remained and the banding provided an outlet diameter of ~10-12 mm. The laparoscopic adjustable gastric banding (LGB) technique involved placing a silicon inflatable gastric band horizontally around the proximal part of the stomach; a pouch was therefore created by inflating the band via a subcutaneous port.

Gastric bypass (GB) was performed using the Rou-en-Y technique. This is a combined restrictive and malabsorptive technique. A restrictive gastric pouch was created and separated from the remainder of the stomach. The continuity was then restored with a Roux-Y-limb, which was connected to the jejunum. The sensation of fullness is created by food entering the gastric pouch. This food then enters the jejunum via the Roux-Y-limb. The length of the common limb determined the degree of malabsorption.

## Results

129 cases were identified in the time period 1990-2004. 85.3% (n = 110) were female and 14.6% (n = 19) were male. Procedures undertaken on the 129 patients studied are listed in Table 2 with the number of patients in each study group. The majority of patients underwent vertical band gastroplasty.

**Table 2:** Bariatric Surgical Interventions carried out at the Manor Hospital Walsall 1990-2004.

**Bariatric Intervention**	**Number of patients n = 129**	**% of Study population**
Vertical Band Gastroplasty (VBG)	105	81.4

**Laparoscopic gastric banding (LGB)**	6	4.6

Gastric bypass (BG)	18	14

Table 3 shows the most common post-operative complications according to procedure undertaken. Of note, there was no mortality.

**Table 3:** Most Common Post-operative Complications as per Bariatric Surgical Intervention

**Bariatric Surgical Intervention**	**Complication**	**% of patients affected**
Vertical Band Gastroplasty	Stenosis	28%
	
	Bowel obstruction	9%
	
	Hernia	9%
	
	Vomiting (but no obstruction)	7%
	
	Staple disruption	2%

Gastric bypass	Vitamin B12 deficiency	20%

	Hernias	20%

For VBG, 40 out of 105 patients had weight gain by the 5th follow-up visit. This compared with 5 out of 18 patients after the same timescale for the GB group and 1 out of 6 in the LGB group.

Further intervention was required in 44 out of 129 cases. Stenosis in 28 of the 105 VBG patients resulted in oesophagogastroduodenoscopy (OGD) and dilatation; bowel obstruction (n = 9) and breakdown of anastomosis disruption (n = 7) out of the same group necessitated revision of the vertical band gastroplasty and subsequent gastric bypass.

For the gastric bypass group (n = 18), the post operative complications included incisional hernia (2/18), vitamin B12 deficiency (2/18) and superficial wound infection (2/18). In the laparoscopic gastric banding group (n = 6) there was one case of umbilical port access site herniation.

**Figure 1:**
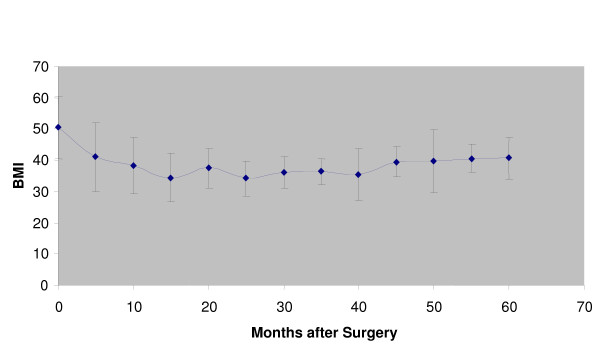
**Vertical band gastroplasty: BMI vs. months after surgery**.

**Figure 2:**
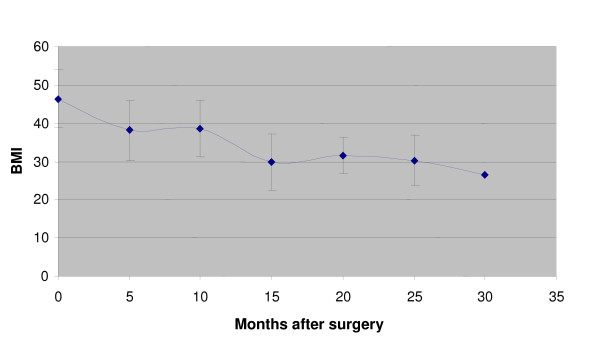
**Gastric bypass: BMI vs. months after surgery**.

**Figure 3:**
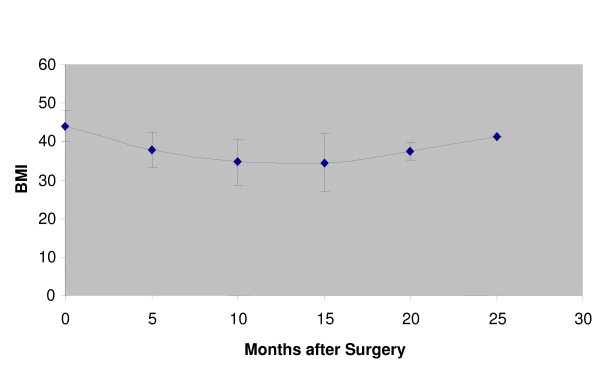
**Laparoscopic band gastroplasty: BMI vs. months after surgery**.

Figures 1, 2 and 3 show the change in BMI for the different interventions over time after surgery.

**Figure 4:**
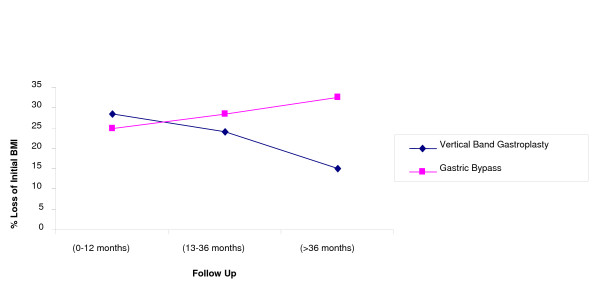
**Loss BMI in the short, medium, and long term for bariatric surgery 1990-2004**.

The percentage loss of initial BMI in the short medium and long term is illustrated in Table 4, Figure 4. There is an increased loss of BMI in the long term in the gastric bypass group (32.5%) when compared to the VBG group (14.97%). There was no regain of weight in patients who underwent gastric bypass, as compared to vertical band gastroplasty (27%), Table 5. There were no significant numbers of patients treated by LGB to enable a satisfactory comparative analysis.

**Table 4:** % Loss of initial BMI in the short, medium and long term for the bariatric surgical procedures undertaken at the Manor Hospital Walsall between 1990 and 2004.

**Procedure**	**% Loss of initial BMI**	
	
	**Vertical Band Gastroplasty**	**Gastric Bypass**
	
		
Short Term (0-12 months)	28.44	24.85

Medium Term (13-36 months)	24.13	28.4

Long term (>36 months)	14.97	32.5

**Table 5:** % of Patients who Regained Weight in the Long Term (>36 months)

**PROCEDURES**	**Gastroplasty**	**Gastric Bypass**
	
	**Vertical Band**	
		
	27	0

## Discussion

Gastric bypass achieves good long term results. A recent long term follow-up study performed by MacLean *et al *defined post-operative success in gastric bypass surgery as a reduction in weight to a BMI <35 kg/m^2^. By this criterion, a successful outcome was achieved in 93% of patients whose initial BMI was less than 50 kg/m^2^, and in 57% of those with an initial BMI greater than 50 kg/m^2 ^[7].

With restrictive procedures, sustained weight loss in the long term is not achieved according to the literature. Of 70 patients who underwent vertical band gastroplasty between 1985 and 1989, only 14 (20%) had experienced a durable loss of >50% of their excess weight [8]. With the laparoscopic adjustable gastric band, results are quite mixed. A French study of 400 patients demonstrated a loss of 50% of excess body weight at 2 years follow-up [9] whilst in America, DeMaria reported on 37 patients and found that these patients lost only 18% of their excess weight at 3-18 months after surgery. >40% of the patients in the DeMaria series had their band removed, most commonly due to inadequate weight loss [10].

The results of up to 10 years of follow-up in the Swedish Obese Subjects Study also make interesting reading. Patients with a BMI of at least 34 (males) and 38 (females) who underwent Bariatric surgery were compared to those who had no surgery. The mean weight losses from baseline in the surgical group were 23% at 2 years and 16% at 10 years as compared to weight gains of <1% and <2% respectively in the control group [11]. At the 10 year follow-up, 'recovery' from diabetes, hypertension, hypertriglyceridaemia, and hyperuricaemia (but not hypercholesterolaemia) was significantly more likely in the surgery group than in the control group; in addition, the new development of diabetes, hypertriglyceridaemia, and hyperuricaemia was less common in the surgery group [12].

Our study shows that Bariatric surgical procedures are relatively safe to undertake in view of the well established benefits. For vertical band gastroplasty, which had the largest cohort of patients, there was an initial reduction from the initial BMI for the patient population after surgery but this weight loss is not maintained, with regain of weight after ~40 months post-operatively (Figure 1). The reasons for this we hypothesise is that we feel these patients have a psychological need to overeat; the neostomach post-operatively initially mechanically restricts them from this but over time, the neo-stomach expands to accommodate this need to eat more and hence they regain weight. These results and the reasons for it are comparable to that for laparoscopic adjustable band gastroplasty.

Gastric bypass has the best results in this particular series (Figure 2). Weight loss is maintained in the long term and there is no regain of weight. The combined restrictive and malabsorptive components of this intervention appear to be the most effective intervention for weight loss in the long term.

We propose that further research in our centre is carried out into Bariatric surgery in terms of the health benefits i.e. the resolution of diabetes and sleep apnoea syndrome as well as the improvement in hypertriglyceridaemia and hypertension.

In conclusion, Bariatric surgery is safe with short to medium term weight loss being achieved. Gastric bypass is the most effective intervention and is currently being offered as the senior author's primary intervention.

## Competing interests

The authors declare that they have no competing interests.

## Authors' contributions

TO was Responsible for data collation, analysis and draft of the manuscript. MS was responsible for the propagation of the study, applied and gained local audit department approval, and supervised the data collation and manuscript draft. AK was the senior author responsible for all procedures in the series of patients described and was responsible for supervision of the study from instigation to completion. All authors read and approved the final manuscript.
